# A novel spine tester TO GO

**DOI:** 10.1002/jsp2.70002

**Published:** 2024-10-27

**Authors:** Jan Ulrich Jansen, Laura Zengerle, Marcel Steiner, Vincenza Sciortino, Marianna Tryfonidou, Hans‐Joachim Wilke

**Affiliations:** ^1^ Institute of Orthopaedic Research and Biomechanics Centre for Trauma Research, University Hospital Ulm Ulm Germany; ^2^ Veterinary Medicine, Clinical Sciences Utrecht University Utrecht Netherlands

**Keywords:** 3R principle, animal testing, ex vivo, range of motion, spine tester

## Abstract

**Background:**

Often after large animal experiments in spinal research, the question arises—histology or biomechanics? While biomechanics are essential for informed decisions on the functionality of the therapy being studied, scientists often choose histological analysis alone. For biomechanical testing, for example, flexibility, specimens must be shipped to institutions with special testing equipment, as spine testers are complex and immobile. The specimens must usually be shipped frozen, and, thus, biological and histological investigations are not possible anymore. To allow both biomechanical and biological investigations with the same specimen and, thus, to reduce the number of required animals, the aim of the study was to develop a spine tester that can be shipped worldwide to test on‐site.

**Methods:**

The “Spine Tester TO GO” was designed consisting of a frame with three motors that initiate pure moments and rotate the specimen in three motion planes. A load cell and an optical motion tracking system controlled the applied loads and measured range of motion (ROM) and neutral zone (NZ). As a proof of concept, the new machine was validated and compared under real experimental conditions with an existing testing machine already validated employing fresh bovine tail discs CY34 (*n* = 10).

**Results:**

The new spine tester measured reasonable ROM and NZ from hysteresis curves, and the ROM of the two testing machines formed a high coefficient of determination *R* ^2^ = 0.986. However, higher ROM results of the new testing machine might be explained by the lower friction of the air bearings, which allowed more translational motion.

**Conclusions:**

The spine tester TO GO now opens up new opportunities for on‐site flexibility tests and contributes hereby to the 3R principle by limiting the number of experimental animals needed to obtain full characterization of spine units at the macroscopic, biomechanical, biochemical, and histological level.

## Graphical abstract

Biomechanical testing after spinal animal experiments is often neglected because testing machines for flexibility measurement are large and difficult to transport, and the same specimen can only be used ex vivo for either biomechanics or biology. With the help of a new portable spine tester, the same specimen can be used for both biological and biomechanical testing, avoiding additional animals and improving the 3R principle. The machine has been validated for future use in any lab all over the world.

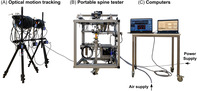

## 1 INTRODUCTION

Low back pain (LBP) is the leading cause of disability and morbidity worldwide and has affected 619 million people globally in 2020 alone.^1^, ^2^, ^3^, ^4^ The cause of LBP is multifactorial, but it is widely accepted that intervertebral disc (IVD) degeneration is a major contributor to LBP and represents a high economic burden.^5^, ^6^, ^7^ Novel therapeutic ideas for disc degeneration include (stem‐) cell therapy, biomaterial injection, and systemic therapies.^8^, ^9^, ^10^, ^11^ Hence, an increasing number of biomaterials are showing promising results in vitro and ex vivo, and preclinical in vivo studies represent the next step.^10^, ^12^ Mostly animal experiments are performed with rats or mice, but also with larger animals such as rabbits, dogs, pigs, goats, and sheep.^12^, ^13^, ^14^ A recent review article by Lee et al. has highlighted the biomechanics as an essential functional outcome of such new therapies but also that, so far, it has been very difficult to incorporate biomechanical testing within large animal studies using dog, pig, goat, and sheep.^14^ Especially, these large animal studies allow comprehensive measurement of biomechanical properties, which can be translated to human IVD health and disease.^12^, ^14^ However, as only 20% of the papers included in the review by Lee et al. measured biomechanical outcomes, the important parameter “biomechanics” appears to be unexploited in large animal studies in vivo. But when measured, apart from the compression stiffness of the functional spinal units (FSU), flexibility expressed by the neutral zone (NZ) and the range of motion (ROM) are the common parameters used to evaluate the biomechanics of the spine. The NZ describes the range in which the specimen can move freely without external forces, that is, the laxity of the specimen.^15^, ^16^, ^17^ The ROM is the sum of the NZ and the range in which the specimen moves only due to applied loads (also called the elastic zone). Both are another indirect measure of the disc deformation and linked to degeneration and regeneration processes^12^, ^16^ and can also indicate the vulnerability of the IVD.^18^ Hence, quasi‐static flexibility tests in flexion–extension (FE), lateral bending (LB), and axial rotation (AR) with pure moments have developed as the standard testing method to understand the biomechanical effects of surgical interventions, inserted implants, biomaterials, or degenerative changes in spines.^17^, ^19^, ^20^, ^21^, ^22^, ^23^ Several apparatuses exist to study the biomechanics of spines and focus on different testing features.^19^, ^24^, ^25^, ^26^, ^27^, ^28^, ^29^ The existing machines for testing ROM^19^, ^28^, ^29^ are complex and immobile and the transportation to other labs is difficult, if not impossible, and this is a strong limitation for the following reasons. As soon as biomechanical testing has to be performed in animal experiments and no suitable testing machine is available locally, frozen specimens have to be transported to the biomechanical testing machines. The repeated freezing and the long period of time result in tissue changes and damages, which limit the subsequent use of the specimens for histological processing, let alone processing for the purpose of obtaining data at the biochemical and biomolecular level providing scientists with the necessary understanding of what happens at the tissue level with respect to the matrix and the mode of action of the therapy being studied. Therefore, the number of animals and specimens is usually doubled in order to have enough specimens for biomechanical and biological tests. However, this is in direct contradiction to the reduction of animal suffering and hinders animal welfare as part of the 3Rs principle: replacement, reduction, and refinement.^30^ By designing and building a testing machine that can be easily transported to other laboratories and enables equivalent precise measurement compared to existing testing machines, it would be possible to perform both biomechanical and biological investigations with the same specimen and, thus, to reduce the number of animals needed for testing.

The goal of this paper is to introduce the design and validation of a new portable spine tester for in vitro/ex vivo biomechanical testing of animal FSUs, which can be brought to any place in the world.

## 2 MATERIALS AND METHODS

### 2.1 Requirements for a portable spine tester

A list of requirements (Table 1) served as the basis for the development of the new machine. Furthermore, this list will be used in the discussion section as a reference for evaluating the work.

**TABLE 1:** List of requirements for a portable spine tester.

#	Name	Description
1.	Transportability	Lightweight and slim design that fits through all common doors.
2.	Autonomy	No dependence on other components that cannot be transported.
3.	Specimens	Testing of mono‐ and poly‐segmental animal specimens of medium to large species (length 5–40 cm) for which testing the ROM with moments in the range of ±0.5–5 Nm is reasonable, for example, cattle, sheep, goat, and dog.
4.	Boundary loading	Measurement of the ROM with pure moments up to ±5 Nm.
5.	Boundary motion	Measurements of three motion planes (FE, LB, and AR) without re‐clamping the specimen.
6.	Testing protocol	Measurements according to previously established standards (Wilke et al.), for example, a speed of 1°/s and multiple measurement cycles (i.e., preconditioning).^17^, ^19^
7.	Software	Automatic and software‐based machine controlling.
8.	Futurity	As far as possible, adaptability of the machine, for example, for testing higher torques.

Abbreviations: AR, axial rotation; FE, flexion–extension, LB, lateral bending; ROM, range of motion.

### 2.2 Technical features of the designed machine

#### 2.2.1 Concept and metal frame

**FIGURE 1:**
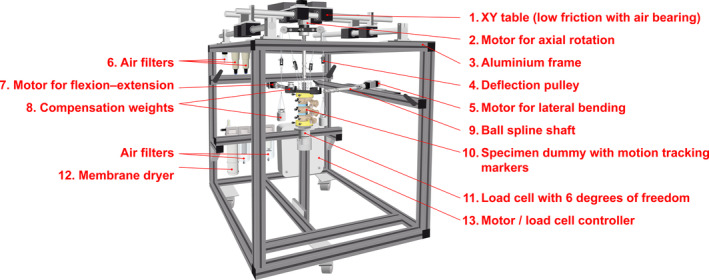
CAD drawing of portable spine tester with all main components: xy table (low friction with air bearing), motor for axial rotation, aluminum frame, deflection pulleys, motor for lateral bending, air filters, motor for flexion‐extension, compensation weights, ball spline shafts, specimen dummy with motion tracking markers, load cell with 6° of freedom, membrane dryer, and motor/load cell controller.

The portable spine tester machine consisted of an aluminum frame (item Industrietechnik GmbH, Solingen, Germany; Figure 1: 3), which held the main components and defined the external dimensions (79.5 cm × 79.5 cm × 115 cm), as well as provided the testing space for the specimen. With a relatively low weight of about 82 kg and wheels, the machine could be moved, transported, and, due to the width of 79.5 cm, it could be moved through any standard door. The frame contained an air bearing xy‐stage, air processing, a force–torque load cell, a digital measuring amplifier, a power supply, motor controllers, and three direct current (DC) motors. Only one motor at a time applied pure torques in one motion plane (FE, LB, or AR) up to a predefined torque limit, and an external motion tracking system recorded the movements of the specimen. From this, the flexibility could be determined. The machine could be stored in a custom‐made transport box and shipped via van or plane all over the world.

#### 2.2.2 Specimen fixation

On the lower side of the cuboid‐shaped machine, the load cell and the caudal part of the specimen were fixed firmly, that is, rigid for all six degrees of freedom, and, depending on the dimensions of the specimen, it could be shifted in all three translations before testing (Figure 1: 10,11). The fixation of the specimen was realized by embedding with polymethylmethacrylate (PMMA, Technovit 3040, Heraeus Kulzer GmbH, Germany) and anchoring flanges to the PMMA via screws. The cranial end was screwed to a lightweight flange, which was weight‐compensated via pulleys and counterweights. At this upper flange, three ball spline shafts directed the rotations of three DC motors for *x*, *y*, and *z* into the specimen free from bending moments or shear forces. Rotations in *x* and *y* for FE as well as LB led to translation of the upper, cranial end of the specimen, which had to be allowed technically to avoid constraining forces.

#### 2.2.3 Weight compensation and air bearing

The weight compensation, as well as the motor for the *z*‐rotation, was installed on air bearings freely movable in *x* and *y* (Figure 1: 1,2). The weight compensation took exactly the weight of the flange (260 g) and the ball spline shafts with the help of the two finely adjusted counterweights via a double‐balanced pulley made of nylon thread (fire line 0.19 mm, Pure Fishing Inc., Spirit Lake, USA) (Figure 1: 4,8). This ensured that the upper flange could be rotated freely around all three directions of rotation without affecting the measurements. The air bearing (∅25 mm) consisted of eight air bearing bushings (S302502, IBS Precision Engineering, Eindhoven, Netherlands, Figure 1: 1)—four per direction—allowing *x* and *y* translations by sliding on four hardened shafts (SSFJ25‐767, MISUMI Europa GmbH, Frankfurt, Germany). The porous bushings made of carbon required a constant air pressure of 4.1 MPa, an air purity of class 3 (ISO 8573‐1^31^), as recommended by the manufacturer, resulting in a 4 μm air gap and zero static friction for the xy‐stage. Air purity was realized with a filter cascade of five different fine filters (AFF2C‐F02D‐T, AM150C‐02D‐T, AF40‐F04‐A, AFM40‐F03‐A, and AFD40‐F03‐A, SMC Deutschland GmbH, Egelsbach, Germany), a pressure regulator (NEW‐1385, IMPLOTEX GmbH, Wurmberg, Germany), and a membrane dryer (IDG30LA‐F03, SMC Deutschland GmbH) (Figure 1: 6,12). The diameter and number of bearings were designed for maximum transmittable torques of ±7.5 Nm in all three rotational directions.

#### 2.2.4 Actuators and load cell

**FIGURE 2:**
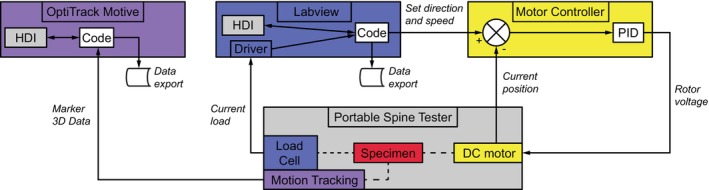
Control loop configuration of the apparatus with Human‐Device‐Interfaces (HDI) displayed for just one actuator and testing direction.

The three brushless DC motors (XBP042713‐03, Ott GmbH & Co. KG, Deißlingen, Germany) were controlled by three motor controllers (AMI1060‐01, Ott GmbH & Co. KG) and connected via controller area network bus with custom‐made Labview software addressed via text commands (Figure 1: 2,5,7 and Figure 2). The actuators were set‐up with an empirical controller design. The maximal torque of the motors equaled 7.5 Nm and was forwarded to the cranial flanges with ball spline shafts (2LT6XUUCL, REIFF Technische Produkte GmbH, Reutlingen, Germany) limited to 2.77 Nm. The six‐component load cell (K6D40, ME‐Meßsysteme GmbH, Hennigsdorf, Germany, Figure 1:11) was placed directly below the specimen and was able to measure reaction forces up to ±200 N (*z* axis) and ± 50 N (*x*‐/*y*‐axis), as well as moments up to 5 Nm (all axes), and communicated with the Labview software via a measuring amplifier GSV‐8DS (ME‐Meßsysteme GmbH). A device‐specific custom calibration of the load cell was carried out by the manufacturer according to DIN EN ISO/IEC 17025, DIN EN ISO 9001, and DIN EN ISO 10012 and stored digitally in the measuring amplifier. The manufacturer was instructed to use typical torque levels for the calibration, which are usually needed for ROM measurements: ±1, ±2.5, ±3.75, and ±5 Nm.

#### 2.2.5 Controlling and software

A custom coded Labview program connected to the load cell amplifier and displayed permanently the current moments and forces (Figure 2). To increase the user‐friendliness, the program guided the user step by step through the measurement. The steps were the following: (1) First setting to zero the forces and moments without specimen; (2) entering the specimen data; (3) moving the motors for zeroing the moments with specimen; (4) performing the test; (5) automatic data export. Text commands sent from the Labview program to the motor controllers, preset target values (axis, speed, and direction), which the motor controller converted into displacement‐controlled actuation of the DC motor with its own loop (Figure 2).

#### 2.2.6 Motion tracking

**FIGURE 3:**
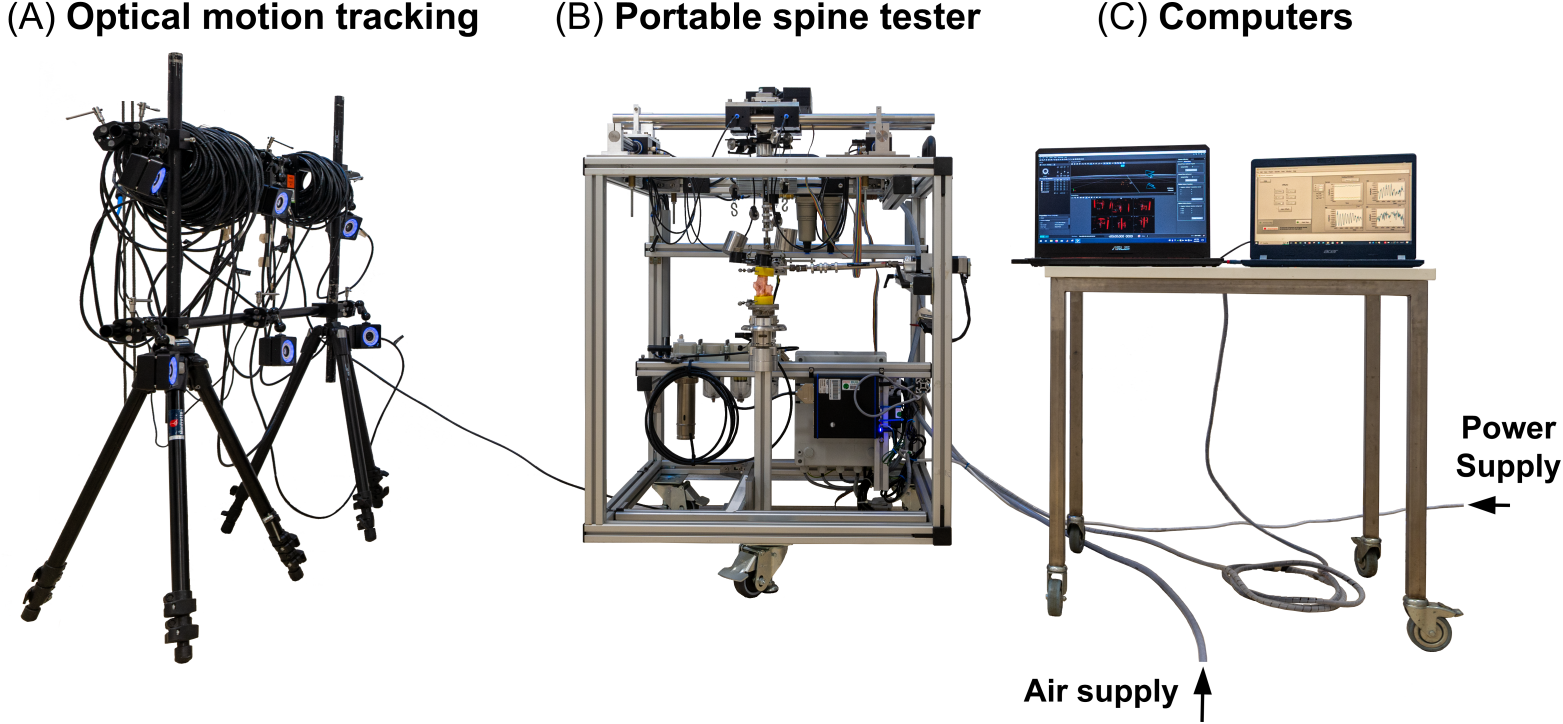
Test set‐up consisting of (A) optical motion tracking system, (B) novel portable spine tester with bovine segment CY3/4, and (C) computers with measurement software (OptiTrack Motive and custom‐made Labview program). Air and power supply are indicated by arrows.

In theory, motion analysis for the novel portable spine tester could be performed with any system and was done externally (Figure 3A). Due to the lightness, OptiTrack with six tracking cameras (Prime13, OptiTrack, Oregon, USA) with a specific developer tool (Motive 4.0, OptiTrack) was used for motion tracking. Three markers per vertebra were screwed on the specimens' PMMA. Following successful calibration in accordance with the manufacturer's guidelines and a spatial mean error of 0.13 ± 0.01 mm, the global coordinate system of the motion analysis system was superimposed on the coordinate system of the portable spine tester (and thus also on the coordinate system of the specimen). Motion sequences recorded the 3D position of the markers for the entire flexibility test per axis. In the software, three markers per vertebra were each assigned to a rigid body, and then the rotation was calculated in the global coordinate system so that each rotational motion about *x*, *y*, and *z* of each vertebral body could be determined. This ensured accurate evaluation of the ROM in the exact anatomical axes of the specimen. After exporting the kinematic parameters of each rigid body, customized software (Matlab, The MathWorks, Massachusetts, USA) was used to calculate the angle differences between the vertebral bodies, and the motion and moment data were merged in order to calculate the hysteresis. In this way, ROM and NZ were determined. The Optitrack motion analysis system was recalibrated for each specimen.

#### 2.2.7 Transportation

**FIGURE 4:**
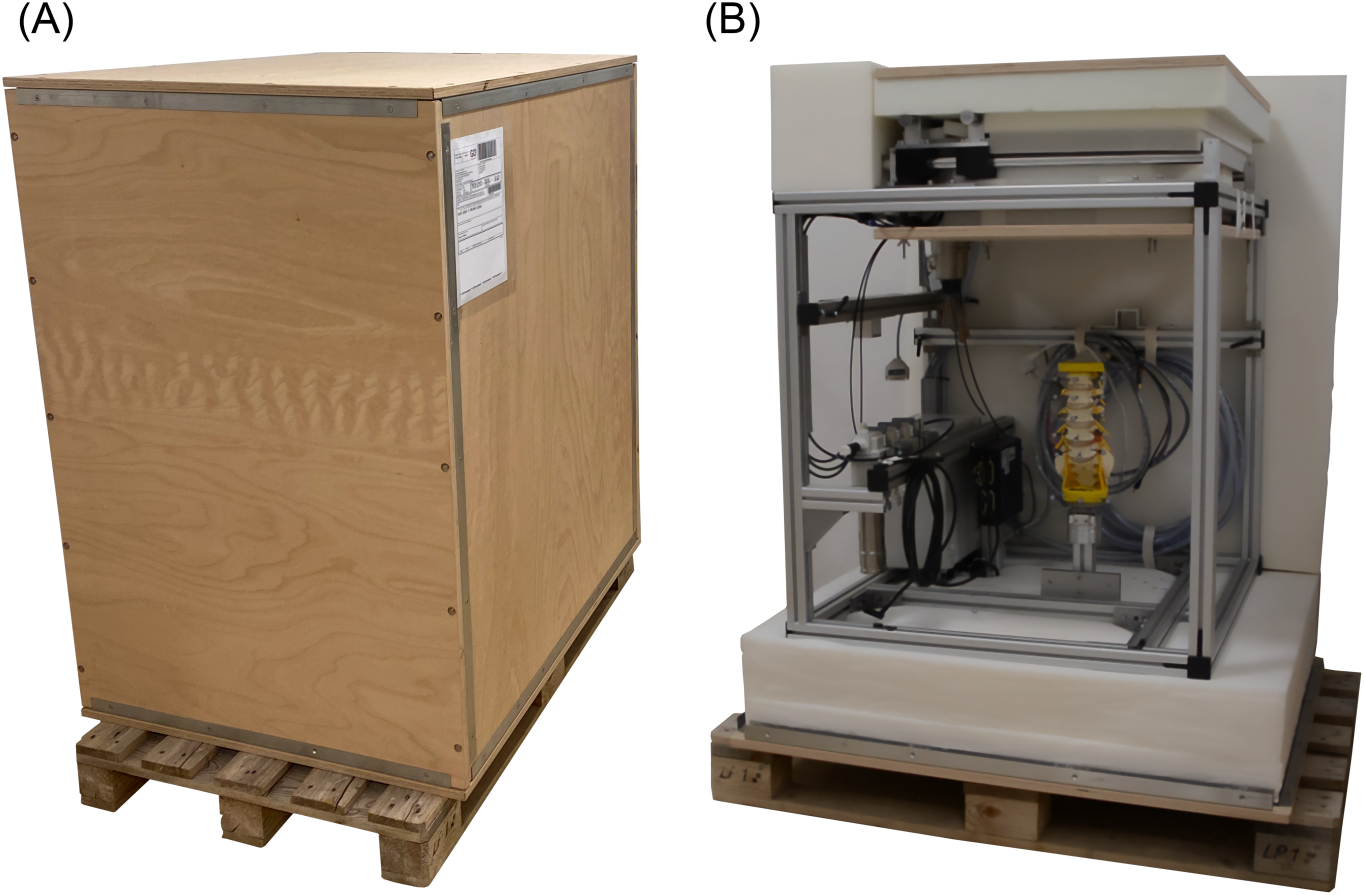
(A) Custom‐made box (104 cm × 89 cm × 135 cm) on pallet for the transportation of portable spine tester to other labs. (B) Open transport box with portable spine tester and special cushioning for air bearing.

The main requirement of the machine was that it could be transported wherever necessary. For shipment to other laboratories worldwide, a pallet‐based transport box (104 cm × 89 cm × 135 cm, Figure 4A) made of solid multiplex boards (thickness of 2 cm) was designed, which especially protected the air bearing by special cushioning (Figure 4B). Polyurethane packaging foam (volume weight = 25 kg/m^3^, compression strength = 4.5 kPa) was used for cushioning. The outer dimension was chosen so that the transport box could be moved indoors (<90 cm). Furthermore, the box was constructed to be demountable into its individual parts.

### 2.3 Existing machine as comparison

**FIGURE 5:**
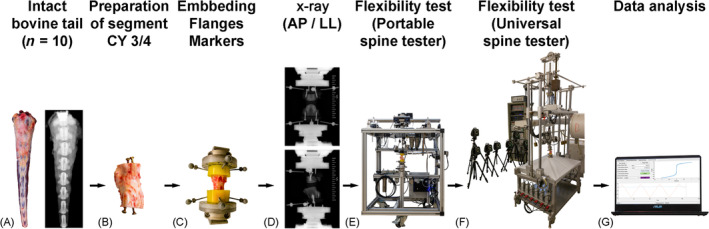
Steps of the experiment (from left to the right): (A) Bovine tails fresh from the slaughterhouse (*n* = 10), directly checked for abnormalities with x‐ray. (B) After preparation of only segment CY3/4, (C) embedding in PMMA, fixation of flanges, and motion tracking markers. (D) x‐ray in antero‐posterior and latero‐lateral plane. (E) Flexibility test with the novel portable spine tester in flexion–extension (FE), lateral bending (LB), and axial rotation (AR). (F) Flexibility test with the existing universal spine tester in FE, LB, and AR. (G) Data analysis and statistics.

The universal spine tester by Wilke et al. was used to compare the results of the new machine (Figure 5F).^19^ The machine was equipped with a 6 degree of freedom (DOF) load cell (FT 1500/40, Schunk GmbH & Co. KG, Lauffen am Neckar, Germany) with a maximal torque limit of 40 Nm and a preciseness of ≤1%. Motion tracking was conducted with the Vicon MX13 system (Vicon Motion Systems Ltd., Oxford, UK) using 10 cameras and three markers per vertebra, as it has been used for many different previous spinal flexibility tests, for example, by Volkheimer et al., Liebsch et al., and Zengerle et al.^32^, ^33^, ^34^

### 2.4 Experimental

#### 2.4.1 Testing procedure with bovine specimens

For the validation, we did not use specimens from sheep, dogs, or other small animals, but bovine tail segments of comparable size, as these are easy to obtain in fresh condition in unlimited numbers and at low cost without ethical concerns. A total of 10 bovine mono‐segmental tail motion segments (male cattle, age 12–24 months, segment CY34) were obtained from a local slaughterhouse (Ulmer Fleisch, Ulm, Germany) to compare the new portable spine tester with the existing machine. No ethical approval was required. Prior to preparation, the specimens were screened for signs of anatomic anomalies, growth abnormalities in the vertebral bodies, previous vertebral fractures, and degenerative changes using anterior–posterior x‐ray (DR Panel PIXX1417, PIXXGEN Corporation, Gyeonggi‐do, Korea) (Figure 5A). After removing muscles and ligaments, the segments of level CY3/4 were cut out through the middle of the adjacent vertebra CY3 and CY4 (Figure 5B) and embedded in PMMA cranially and caudally. For testing, flanges were screwed on the hardened PMMA blocks on both sides, and motion tracking markers were attached via custom marker screws (Figure 5C). x‐rays were taken in antero‐posterior and latero‐lateral view of the embedded specimen with flanges and markers (DR Panel PIXX1417; Figure 5D). The biomechanical in vitro tests were first performed with the novel portable spine tester (Figure 5E) and afterwards with the universal spine tester,^19^ both according to the testing criteria by Wilke et al.^17^ (Figure 5E/F). In both machines, pure moments of ±1 Nm were applied without preload in the three anatomical planes: FE, LB right and left, and AR left and right. The motor speed was set to 1°/s in each loading direction. Both machines allowed the specimens to move unconstrained in the five uncontrolled DOF during loading. All specimens were loaded with three and a half cycles in each motion plane.^17^ The first two cycles were applied for preconditioning, and the third cycle was used for evaluation at ±1 Nm (Figure 5G). NZ was calculated using the zero‐load method.^17^, ^35^, ^36^

#### 2.4.2 Evaluation, data analysis, and statistics

First, the performance of the new machine was evaluated qualitatively with respect to the above requirements for transport, applicability, and resulting hysteresis curves. Then the results of both machines were examined quantitatively. The data was processed using Matlab (The MathWorks) and Excel 16 (Microsoft Corporation, Redmond, USA). Normal distribution was assessed with the Shapiro–Wilk test (IBM® SPSS® Statistics Version 29; IBM Corp., Armonk, USA). For both ROM and NZ, significant differences between the testing machines were analyzed using the non‐parametric Wilcoxon test. Furthermore, the ROM data points of all motion planes were analyzed and statistically assessed using a linear regression model after the dataset has been tested for the requirements (see Section 3.4). Differences were considered significant for *p* < 0.05.

## 3 RESULTS

### 3.1 Design and transport

Within a first ex vivo study with fresh bovine tail specimens, the new portable spine tester enabled the determination of ROM and NZ of 10 different intact bovine specimens CY3/4 with small torques of up to ±1.0 Nm (currently limited by the ball splines to ±2.5 Nm) in three motion planes. The portable spine tester successfully loaded the specimens and controlled the 3.5 cycles of the test. OptiTrack motion analysis was used for measuring the motion of the specimens. First tests showed the safe packability of the transport box (Figure 4) and that transportation with a lifting cart or a van could be easily performed. Setting up the machine and the motion tracking system with calibration was done within 2 h. Set‐up and measurement were conducted by qualified personnel.

### 3.2 Hysteresis curves

**FIGURE 6:**
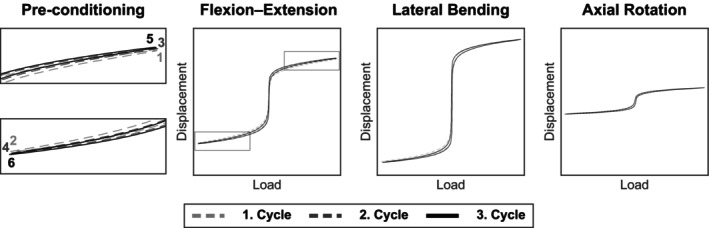
Exemplary hystereses of flexion–extension (FE), lateral bending, and axial rotation in load–displacement‐coordinates for the portable spine tester. The first three cycles are displayed with three different line types according to legend. The graphs left show enlarged ends of the FE hysteresis and point out the typical preconditioning behavior of visco‐elastic materials.

Moment‐angle diagrams are characterized by the typical S‐shape hysteresis as well as the ROM, NZ, and elastic zone areas for all motion planes (Figure 6). A noticeable feature was an elongated NZ, which had a very narrow width, resulting in a small area of the hysteresis curve. This feature was more prominent for FE and LB than for AR. According to Di Pauli Von Treuheim et al., the hysteresis curves represent more likely a triphasic than a visco‐elastic profile.^35^ With each new cycle, the displacement became slightly larger—both in negative and positive direction, indicating that a preconditioning effect was observed.

### 3.3 Absolute range of motion and neutral zone

**FIGURE 7:**
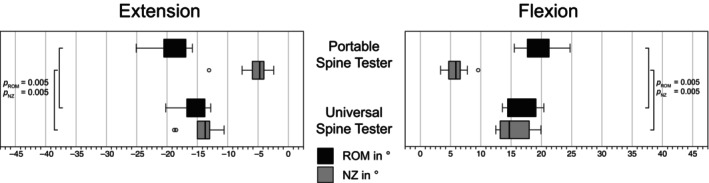
Range of motion (ROM) and neutral zone (NZ) of motion segments from the bovine tail (CY3/4) in flexion‐extension loaded with pure moments of ±1 Nm tested with the novel portable spine tester and the universal spine tester (Wilke et al.^17^). *p*_ROM_ and *p*_NZ_ describe the differences within ROM and the NZ, respectively, using a Wilcoxon test. Circles indicate outliers.

**FIGURE 8:**
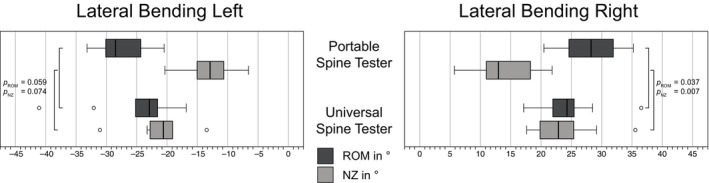
Range of motion (ROM) and neutral zone (NZ) of motion segments from the bovine tail (CY3/4) in lateral bending left/right loaded with pure moments of ±1 Nm tested with the novel portable spine tester and the universal spine tester (Wilke et al.^17^). *p*_ROM_ and *p*_NZ_ describe the differences within ROM and the NZ, respectively, using a Wilcoxon test. Circles indicate outliers.

**FIGURE 9:**
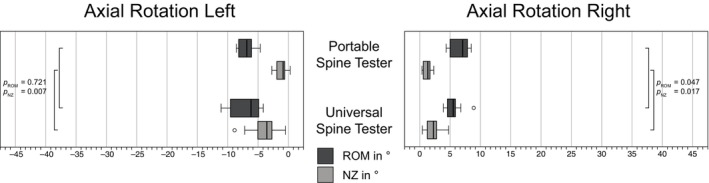
Range of motion (ROM) and neutral zone (NZ) of motion segments from the bovine tail (CY3/4) in axial rotation left/right loaded with pure moments of ±1 Nm tested with the novel portable spine tester and the universal spine tester (Wilke et al.^17^). *p*_ROM_ and *p*_NZ_ describe the differences within ROM and the NZ, respectively, using a Wilcoxon test. Circles indicate outliers.

The Shapiro–Wilk test indicated a higher rate of violations of the normality assumption (12.5%), therefore, non‐parametric tests were used. Values were specified as median and statistical differences tested using Wilcoxon. For both spine tester, the highest ROM values were found for LB, followed by FE and AR (Figures 7, 8, 9). For ROM in FE, the portable spine tester measured −18.1° and 18.4° versus −14.8° and 16.7° for the universal spine tester, with significant differences (*p* = 0.005). For LB, ROM measured by the portable spine tester averaged to −28.5° and 28.2° versus −22.9° and 24.3° by the universal spine tester (*p* ≤ 0.059). ROM in AR was −6.8° and 7.1° for the portable spine tester and −3.5° and 2.3° for the universal spine tester with significant differences for AR right (*p* = 0.047) but not for AR left (*p* = 0.721). Regarding NZ in FE, the portable spine tester measured significantly lower ranges (−4.7° and 5.9°) than the universal spine tester (−13.7 and 14.7) (*p* = 0.005). Similar for LB, the portable spine tester determined NZ medians of −13.0° and 13.0° versus the universal spine tester with −20.6° and 22.9° (*p* = 0.074 for left, *p* = 0.007 for right LB). Finally, for AR, the portable spine tester obtained significantly lower NZ ranges (0.9° and 1.3°) than the universal spine tester (−3.5° and 2.3°) (*p* ≤ 0.017). Overall, for all motion planes and for the low moment values relevant for smaller animals like dogs, the portable spine tester measured slightly higher ROM and lower NZ than the existing universal spine tester. Since the total of the absolute values of all individual specimens was compared here and not the machines among each other for each specimen itself, a linear regression analysis follows in the next section.

### 3.4 Regression analysis of total ROM: Portable spine tester versus universal spine tester

**FIGURE 10:**
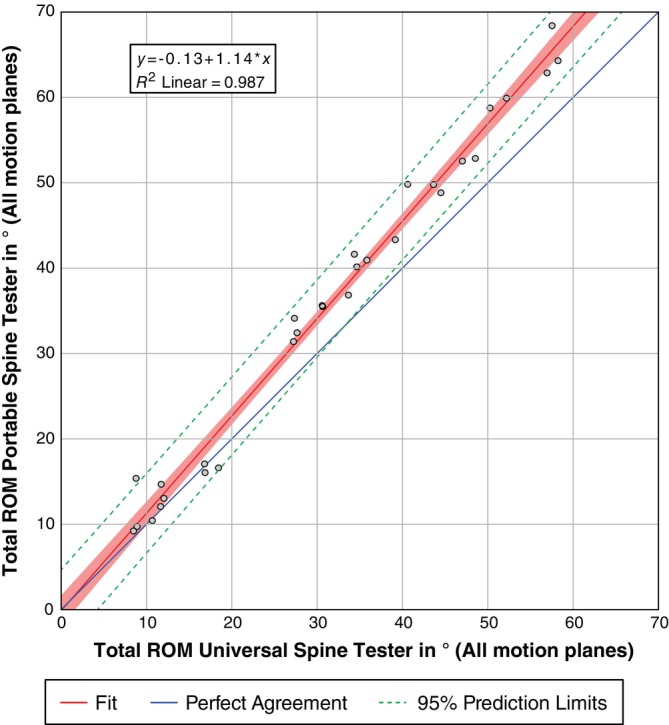
Regression analysis of total range of motion (ROM) in three motion planes for both spine testers, showing the values of the portable spine tester (*y* axis) versus values of universal spine tester (*x* axis) as the ground truth. The 95% confidence interval of the correlation (green dashed lines) and the 95% confidence limits of the regression line (rendered in solid light red) as well as the line indicating a perfect correspondence between both spine testers (in blue) are shown.

To further elucidate the comparability of the new and the existing machine regarding the ROM of the same specimen, the total ROM data of the machines were correlated with each other using the data of all three motion planes (Figure 10). All necessary conditions for the linear regression model were tested, and neither auto‐correlation (Durbin‐Watson: 2.047), strong outliers, nor deviations from the normal distribution of the residuals (Shapiro–Wilk: *p* = 0.780) were found. The *R* ^2^ for the overall model was 0.987 (adjusted *R* ^2^ = 0.986), representing a high goodness‐of‐fit. The portable spine tester was able to measure ROM values that correlate statistically significantly with those of the universal spine tester (*F* (1, 28) = 2100.976, *p* < 0.001).

## 4 DISCUSSION

This paper introduces a new custom‐designed, portable spine tester that can measure flexibility of mono‐ and poly‐segmental spinal specimens in three motion planes under quasi‐static conditions. With its dimension and weight (79.5 cm × 79.5 cm × 115 cm; 82 kg), it can be transported, which is not possible with the existing universal spine tester by Wilke et al.,^19^ which is ca. three times heavier and spans dimensions of 125.0 cm × 80.0 cm × 245.0 cm. In its current development stage, the new machine is suitable for testing animal spines with pure moments of up to ±2.5 Nm (validated at ±1.0 Nm) and angle ranges of at least 65° and more, whereas the universal spine tester can measure with moments up to ±40.0 Nm. The portable spine tester is characterized by applying pure moments without bending forces, its special transportability, quick assembly on‐site, low friction in the xy‐translation, and a very precise load cell. This paper aimed to validate the machine using fresh mono‐segmental bovine tail specimens and demonstrated reproducible and reasonable results in all motion planes with pure moments of ±1 Nm. Validating the portable spine tester with higher moments than ±1 Nm would also be of high interest but cannot be performed with bovine tail segments that can be obtained fresh from the slaughterhouse without ethical concerns. This work shows the successful implementation of the comprehensive list of requirements and presents simulated experiments in the operating room, including transportation, machine set‐up within 2 h, specimen preparation, and embedding with fresh animal tissue.

### 4.1 Comparison with the requirements list

1. The novel spine tester is relatively light, compact, and wheeled and fits through doors both with and without the custom‐made transport box (without box: 80 cm, with box 90 cm); hence, the new machine can be shipped anywhere in the world for experiments.
2. Additional components are necessary for using the spine tester but can also be transported:
  - Compressor if not available, needs to be shipped. Any available compressed air can be used since the high requirements of the air bearing for air purity according to ISO 8573‐1 (particle size <5 μm, humidity <128 ppm. vol., oil <1 mg/m^3^) are covered by the filters on the machine itself.
  - Motion analysis systems such as OptiTrack present no difficulties in transportation due to the small size of the Prime 13 cameras (6.9 cm × 6.9 cm × 5.3 cm).
  - Laptop computers, which are transportable.
3. Despite the small dimensions of the machine, the requirements for the spinal specimen length of 5–40 cm are fulfilled. This allows for mono‐ and poly‐segmental testing since pure moments are applied and optical motion tracking can assess segmental ROM.
4. The aim was to be able to carry out tests with pure moments of up to 5 Nm. The maximal load capacity of the frame, the motors (up to 7.5 Nm), and the load cell (up to 5 Nm) allow this. However, the ball shafts that enable low friction translation perpendicular to the testing plane could only be procured with a maximum load capacity of 2.77 Nm, that is, practically 2.5 Nm. This leads to a deviation from the requirements for the maximum test torque. However, an alternative solution for higher transmittable moments will be developed in the future.
5. With three DC motors and ball spline shafts, the specimen stays with the same orientation in the machine for all motion planes, which is an improvement compared to Goertzen et al.^28^ Furthermore, re‐clamping is not needed. Three motors at the same time also enable complex loading conditions, which represents a very relevant feature for a spine tester to simulate physiological loading conditions.^37^, ^38^, ^39^
6. The device was programmed to follow the testing criteria of Wilke et al.^17^ with repetitions of 3.5 cycles and a testing speed of 1°/s.
7. The customized Labview software enables automatic and software‐based machine controlling and has already integrated the many basic functions as user‐friendly as possible so that the machine might be operated by anyone in the future.
8. The item frame enables new accessories to be attached. For example, the load cell can be replaced by other models from ME‐Systems with a lower or higher measuring range. Other accessories can be water steamers or a water bath for improving the water content of the IVD during testing.

### 4.2 What kind of specimens can be tested

Related to the literature, the moments of up to 2.5 Nm are predominately suitable for testing of cervical spines of human, ovine, and bovine origin, as well as for rabbits, goats, pigs, and canine at all segmental levels.^28^, ^40^, ^41^, ^42^ A future optimization of the ball spline shafts and a load cell for up to 7.5 Nm would improve the machine for testing specimens of larger segment levels of all mentioned species with comparable conditions to the existing literature.^42^, ^43^, ^44^, ^45^, ^46^ If several segments of a spine are to be tested, it is advantageous to test several segments at once as one piece. This is due to the fact that all segments are measured at the same time using the motion analysis system and pure moments, which saves time for clamping and PMMA embedding. This is a very important aspect for 1 day long experiments in the operating room (OR) before the specimens are handed over to colleagues for the biological analysis. In addition, poly‐segmental testing more closely mimics the in vivo situation as certain spinal structures, for example, the longitudinal ligaments, are continuous.^16^ The more segments of the same spine are tested at the same time, the longer the mounted specimen will be. However, the practically usable specimen length seems rather limited by the maximal ROM that can be handled by the machine than by the specimen length: For example, for pure moments of 2.5 Nm, a cervical, ovine spine from C2 to T1 has an accumulated ROM of 41.19° for flexion, 46.00° for extension, 72.10° for LB right, and 32.64° for AR left,^43^ whereas its length sums up to ca. 31 cm.^47^ Compared to the angles measured in this study, more segments could not be tested, although larger specimens than 31 cm would fit into the machine.

### 4.3 Validity of ROM measurement

The ROM and NZ measured with the new portable spine tester have been compared to an existing spine tester to evaluate the validity of the new machine (Figures 7, 8, 9). The new machine measures larger values for ROM than the existing one and also shows a higher scatter of measured values. However, the symmetry of the values for LB left/right and AR left/right is consistent with the results typically found in the literature.^16^, ^41^, ^48^ However, there are several reasons why the two devices may not measure exactly the same values, but still reflect the real situation. Different motion analysis systems, different load cells, and different bearing technologies (air bearing vs. rolling bearing) were used. Any of these points could explain the slight ROM differences between the two machines. Furthermore, the specimens have been tested subsequently, and this could have also impacted the results. In a second step, we correlated the ROM values of both machines and found a significant linear regression with a very high *R* ^2^ (Figure 10). Therefore, we believe that the new machine has proven to measure ROM in a repeatable, accurate, and standardized way. The differences may be particularly noticeable at low moments, where friction and the precise measuring range of the portable spine tester's load cell may have a greater effect. On the other hand, the universal spine tester can perform tests with moments of up to 40 Nm and muscle force simulation, which the portable spine tester could not achieve, even with modification.

### 4.4 Validity of NZ measurement

Distinct differences between the machines occur in the NZ values that describe the laxity or instability of a specimen.^15^, ^17^, ^36^ In this study, a greater NZ was measured for the universal spine tester than for the portable spine tester (Figures 7, 8, 9). Qualitatively, the hysteresis curves of the two testing machines have different widths or areas of the hysteresis curves. The curves of the portable spine tester show a very long vertical, thin NZ (Figure 6), which is characteristic for bovine tails and its curve shape can be attributed to the hysteresis of the triphasic shape.^35^ The NZ was determined using the zero‐load method, in which the vertical distance between the positive and negative phase is measured in the load–displacement curve.^17^, ^35^ This method is widely used, and one advantage of the zero‐load method is that it does not require arbitrary user input.^35^ According to Di Pauli Von Treuheim et al., the zero‐load method is particularly sensitive to degeneration‐induced changes in motion segment instability and is commonly used in larger animal models and in human cadaver studies.^17^, ^32^, ^35^ On the other hand, the zero‐load method may not be ideal for studies with smaller motion segment models due to the smaller dynamic range over which their load–displacement curve runs.^35^, ^49^ Furthermore, the zero‐load method is particularly vulnerable to inconsistent NZ measurements when load–deflection profiles contain long laxity ranges, since a small change in load (*x*‐axis) in one of the two limbs of the hysteresis will cause a large difference in the *y*‐axis intersection and then increase the variability of the calculated NZ magnitude.^35^ The results of the portable spine tester reflect the previously discussed situation with long laxity ranges, and we have to admit as a limitation that the zero‐load method is probably not suitable to evaluate the NZ in a comparable way due to the limitations of the zero‐load method. The evaluation program for the portable spine tester must therefore be adapted, and in future, a different determination method for the NZ must be implemented. In conclusion, similar to the ROM, it can be stated that the machines do not deliver the same values, but the differences can be comprehensibly explained by mechanics and different machine components.

## 5 CONCLUSION

Both spine testers have their key applications for quasi‐static load applications in contrast to other machines for dynamic testing.^24^ The portable spine tester is limited by its design for small moments due to its specialization in flexibility testing but has low friction in xy‐translation and very small dimensions and is therefore suitable for smaller specimens, particularly for smaller species. The existing machine by Wilke et al. is larger and heavier but serves with higher moments and loads as well as more universal functions by allowing the simulation of muscle forces, shear, and more.^19^ With the new portable spine tester, we are taking an important step forward in spinal biomechanics regarding the 3Rs principle and how experiments can be planned and conducted in future. By being able to transport the machine anywhere in the world and to perform flexibility measurements on‐site, the new machine enables spinal animal experiments that more closely follow the 3R principle (namely to replace, reduce, and refine) because the required number of animals can now be reduced. The same animal can now be used for biology and biomechanics, so that after animal testing, the question is no longer: “biomechanics OR biology?” but instead “biomechanics AND biology!” thanks to the “Spine Tester TO GO.”

## AUTHOR CONTRIBUTIONS

All authors contributed to the study conception and design. Material preparation and data collection and analysis were performed by Jan Ulrich Jansen, Laura Zengerle, Marcel Steiner, and Vincenza Sciortino. Hans‐Joachim Wilke created the idea and the concept. Hans‐Joachim Wilke and Marianna Tryfonidou supervised the project, also undertaking project administration and funding acquisition. The first draft of the manuscript was written by Jan Ulrich Jansen and all authors commented on previous versions of the manuscript. All authors read and approved the final manuscript.

## CONFLICT OF INTEREST STATEMENT

The authors declare no conflicts of interest.
